# *Schistosoma mansoni* infection after three years of mass drug administration in Sierra Leone

**DOI:** 10.1186/1756-3305-7-14

**Published:** 2014-01-09

**Authors:** Santigie Sesay, Jusufu Paye, Mohamed S Bah, Florence Max McCarthy, Abdulai Conteh, Mustapha Sonnie, Mary H Hodges, Yaobi Zhang

**Affiliations:** 1 Neglected Tropical Disease Control Program, Ministry of Health and Sanitation, Freetown, Sierra Leone; 2 Helen Keller International, PO Box 352, Freetown, Sierra Leone; 3 Helen Keller International, Regional Office for Africa, Dakar, Senegal

**Keywords:** Schistosomiasis, *Schistosoma mansoni*, Neglected tropical diseases, NTDs, Independent monitoring, Sierra Leone, Mass drug administration, Praziquantel

## Abstract

**Background:**

*Schistosoma mansoni* was moderately-highly endemic in the northeast of Sierra Leone. The national neglected tropical disease control program started mass drug administration (MDA) with praziquantel (PZQ) in six districts in 2009 targeting primary school children only. The effort was scaled-up to seven districts in 2010 targeting school aged children (SAC) and at-risk adults. A cross-sectional sentinel site survey was conducted in 2012 after three rounds of MDA to evaluate the impact of the national program.

**Methods:**

Twenty-six (26) sentinel sites were randomly selected from the baseline mapping survey sites stratified according to the baseline prevalence into high, moderate or low endemic category. Fifty (50) school children (25 males and 25 females) were randomly selected per site. Fresh stool samples were examined in the field using the Kato Katz technique. The results were compared with the baseline data.

**Results:**

Program coverage of 94.8%, 77.1% and 81.7% was reported in 2009, 2010 and 2011 respectively. Independent monitoring in 2011 showed program coverage of 83.9%, not significantly different from the reported result in the same year. The overall prevalence of *S. mansoni* was 16.3% (95% CI: 14.4-18.4%) and mean intensity was 18.98 epg (95% CI: 11.46-26.50 epg) in 2012, representing 67.2% and 85.9% reduction from the baseline respectively. The proportion of moderately and heavily infected children was 3.3% and 1.2%, a significant reduction from 18.2% and 8.8% at baseline respectively.

**Conclusions:**

Sierra Leone has maintained effective MDA coverage with PZQ since 2009. Three rounds of MDA led to a significant reduction of *S. mansoni* infection in the country. In line with the significant progress made in controlling schistosomiasis, the national treatment strategy has been reviewed and MDA will be expanded to include school age children in low endemicity districts with the new national objective for the elimination of schistosomiasis. Sierra Leone is well on its way to eliminate schistosomiasis as a public health problem.

## Background

Schistosomiasis inflicts great health and socioeconomic burdens on the poorest populations in both rural and non-rural settings of the tropics and subtropics, particularly in sub-Saharan Africa [1,2]. It is estimated that over 200 million people are infected with schistosomes worldwide, and schistosomiasis alone results in 200,000 deaths per year in sub-Saharan Africa [1,3]. Infected children that have marginal levels of adequate nutrition are at risk of growth retardation, impaired cognition, and micronutrient deficiencies [4-7]. These factors combine to reduce school attendance and to impair educational performance, which, in turn, lead to a reduction in future productivity and wage-earning capacity [8,9].

The global effort in the integrated control of the neglected tropical diseases (NTDs) currently targets five major ‘tool-ready’ diseases with preventive chemotherapy (PC): lymphatic filariasis (LF), onchocerciasis, schistosomiasis, soil-transmitted helminthiasis (STH) and trachoma [10]. The World Health Organization (WHO) recommends that in highly endemic schistosomiasis communities (prevalence ≥50%), all school aged children (SAC) and ‘at risk’ adults should be treated with praziquantel (PZQ) annually. In moderately endemic communities (prevalence ≥10% and <50%), all SAC and ‘at risk’ adults should be treated biennially. In low endemic communities (prevalence <10%), all SAC should be treated twice during their primary school years [11]. ‘At risk’ adults include all those who have contact with contaminated fresh water sources: fishermen, farmers, alluvial miners, and those households that wash/play in schistosomiasis-contaminated water.

In Sierra Leone, the integrated national NTD control program (NTDCP) started in 2008 with financial support from the United States Agency for International Development (USAID) and technical assistance from Helen Keller International (HKI). One of the original goals of the national NTDCP was to reduce schistosomiasis prevalence in all districts to <10% [12].

Both forms of intestinal and urogenital schistosomiasis have been recognized as endemic in Sierra Leone [13,14]. National mapping in 2008 to 2009 confirmed that the distribution of schistosomiasis was widespread, with moderate-high prevalence in the north-eastern part of the country [15-17]. Mass drug administration (MDA) with PZQ was implemented in seven moderately-highly endemic districts: Kailahun, Kono, Koinadugu, Kenema, Bo, and the north-eastern chiefdoms of Tonkolili and Bombali. In 2009, MDA was piloted in six districts, targeting only school-going children. In 2010, MDA was scaled-up to target all SAC and at risk adults (in this case, all rural adults are at risk) in moderately-highly endemic chiefdoms for seven districts. The treatment frequency within districts varies according to the prevalence level in each chiefdom. Independent monitoring was conducted after MDA to assess drug coverage in 2011. A sentinel site survey was also conducted in 2012 after three rounds of MDA to assess the progress of the national NTDCP. The current paper presents the survey results and discusses the future treatment strategies, in line with the new global objective for the elimination of schistosomiasis.

## Methods

### MDA and data reporting

In Sierra Leone, PZQ was administered by the peripheral health unit (PHU) staff. This decision was made by the NTDCP at the beginning of the program due to the potential side effects of the PZQ treatment in the population that had never been treated. Each year, before MDA, PHU staff receives training or refresher training on PZQ distribution, including use of the dose poles, eligibility of the target population, data recording and reporting, and knowledge and management of potential side effects. Following social mobilization, school-going children received treatment at school-based distribution points, and out-of-school children and ‘at risk’ adults accessed treatment via community-based distribution. PHU staff update the registers for each village in their catchment areas before each MDA and estimate the drug need. They used tally sheets to record the number of PZQ administered during MDA. At the end of MDA, the PHU staff reported the population and treatment numbers to health districts that in turn, collated the data in each district and reported to the NTDCP.

### Independent monitoring on MDA coverage

In July 2011, independent monitoring to assess program coverage, as described previously [18], was conducted seven days after the MDA. At least three villages were sampled per district, with 1 village from a WHO list of consistently low performing villages during immunization campaigns (WHO Sierra Leone, unpublished observation). Independent monitors used simple tally sheets to interview 60 SAC (30 males and 30 females), from classes 5–6 or 12–14 years of age in selected schools, and 60 adults in communities on whether they could recall taking PZQ. Independent monitoring was conducted in the seven endemic districts: Kailahun, Kono, Koinadugu, Kenema, Bo, Tonkolili and Bombali. In the community survey, independent monitors conducted interviews in both public locations and in randomly selected households. The results were compared with the NTDP reported coverage.

### Sentinel site selection

**Figure 1:**
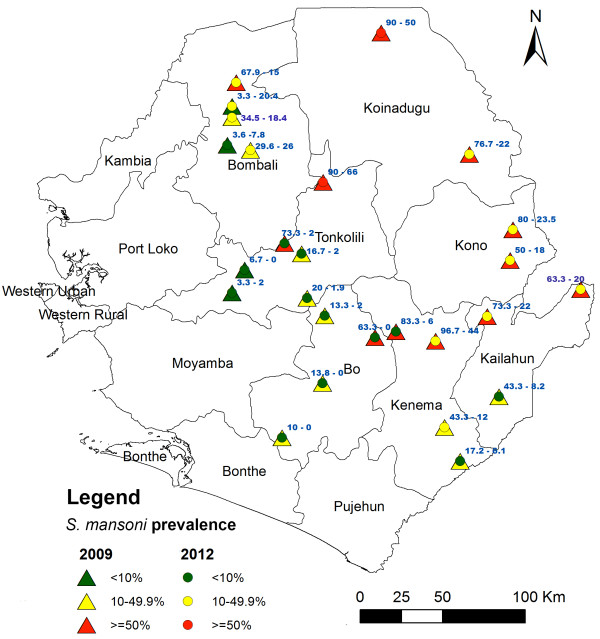
**Sentinel site locations and *S. mansoni *prevalence in Sierra Leone.** Numbers in blue represent the prevalence value at the baseline (the former) and after 3 rounds of MDA (the latter) for each sentinel site.

This study was conducted in February 2012, seven months after the last MDA in 26 sentinel sites in the seven moderately-highly endemic districts. The sentinel sites were randomly selected from the baseline mapping survey sites, which were stratified according to the baseline prevalence into high, moderate or low endemic category. It was intended to select two sites from each endemic category within each district. Details of the baseline surveys have previously been described elsewhere [15,16]. Thus, six sites were selected from Tonkolili district since it had all three prevalence categories, five sites from Bombali, four sites in both Kenema and Bo, three sites from Kailahun and two sites in Kono and Koinadugu districts respectively. In total, 12 sites had high baseline prevalence, 10 sites had moderate baseline prevalence and four sites had low baseline prevalence. The locations and baseline prevalence of each site is shown in Figure 1. In Bombali district, one heavily endemic site received two rounds of MDA (2010 and 2011) while the remaining four moderately endemic sites had received only one round of MDA in 2010. Yoni chiefdom in Tonkolili district had no MDA because of its low prevalence at baseline.

### Sampling and data collection

A total of 50 SAC (25 males and 25 females) aged 10–14 years who were in the upper classes (classes 5–6) were randomly selected from each primary school in each site, according to WHO recommendations [19]. Each child was given a unique ID number, and their name, sex and age were recorded. Once recorded, each child was given one plastic container and asked to provide a fresh stool sample. All stool samples were examined (1 slide per sample) within five hours of collection by the Kato-Katz method and using a 41.7 mg thick faecal smear template [20,21]. All positive slides and 10% of all negative slides were re-examined in the field by the more senior technicians for quality assurance. The number of *S. mansoni* eggs per gram of faeces was calculated by using a multiplier factor of 24.

### Data analysis

Overall, 1,286 children (642 males and 644 females) were examined in the 26 sentinel sites during 2012. The results were compared to the data from the same sites surveyed at baseline (770 children: 373 males and 396 females, with one missing data point for sex). Data were analysed using SPSS statistical software (IBM, version 19). The SPSS Complex Samples Crosstabs was used to calculate the prevalence and the Complex Samples Descriptives was used to calculate the intensity of infection with 95% confidence intervals, taking into consideration the cluster nature of school children using district as strata and school as clusters. Samples were weighted according to the population sizes in the surveyed districts when calculating the overall prevalence and intensity of infection. The arithmetic mean intensity of infection from all the examined subjects (including both positive and negative) was used in the analysis. The individual *S.mansoni* infection was categorized as heavy (≥400 epg), moderate (100–399 epg), and light (1–99 epg) infections [22]. The Chi-square test was used to compare the prevalence, and the Kruskal Wallis test was used to compare the mean intensity of infection between 2009 and 2012. GPS coordinates were recorded and ArcView 10 software (ESRI, Redlands, CA) was used to plot sentinel site locations and prevalence level.

### Ethical considerations

Ethical approval for the survey was obtained from the Ministry of Health and Sanitation, Sierra Leone. Community informed consent was obtained following discussion with District Medical Officers, Chiefdom school inspectors, head teachers and community-teachers associations. All communities included in the sentinel sites were sensitised prior to sample collection. Verbal individual consent was obtained from parents/carers as literacy rates are low in these communities. Children were also given information on the purpose of the activity prior to sample collection.

## Results

### MDA coverage

The reported annual treatment numbers and coverage by district are shown in Table 1. In 2009, targeted individuals were only primary school children, and in 2010 and 2011, the treatment expanded to include SAC both in and out of school and ‘at risk’ adults. Accordingly, 562,980 school children were treated in 2009 with program coverage of 94.8%; 1,831,383 persons were treated in 2010 with program coverage of 77.1%; and 1,781,037 persons were treated in 2011 with program coverage of 81.7%. Specifically for SAC in 2010 and 2011, 403,212 and 540,103 SAC were treated respectively for program coverage of 85.4% and 86.0% in the SAC population accordingly.

**Table 1:** Sierra Leone schistosomiasis treatment numbers and coverage 2009-11

**District**	**2009 reported**			**2010 reported**			**2011 reported**			**2011 IM results**		
	**Targeted**	**Treated**	**%**	**Targeted**	**Treated**	**%**	**Targeted**	**Treated**	**%**	**Interviewed**	**Recalled**	**%**
Bo	-	-	-	278,547	223,096	80.1	183,192	151,887	82.9	660	500	75.8
	(121,467)	(110,300)	(90.8)	(75,679)	(63,545)	(84.0)	(113,500)	(98,196)	(87.0)	(360)	(288)	(80.0)
Bombali	N/A	N/A	N/A	135,705	107,253	79.0	21,810	17,921	82.2	720	625	86.8
				(4,562)	(3,939)	(86.3)	(32,394)	(29,756)	(92.0)	(360)	(314)	(87.2)
Kailahun	-	-	-	417,899	336,014	80.4	438,952	364,249	83.3	1,414	1,138	80.5
	(86,631)	(84,118)	(97.1)	(97,556)	(84,030)	(86.1)	(97,556)	(84,444)	(87.0)	(707)	(599)	(84.7)
Kenema	-	-	-	532,807	416,974	78.3	530,803	423,864	79.9	1,693	1,574	93.0
	(156,496)	(151,203)	(96.6)	(100,439)	(84,868)	(84.5)	(102,443)	(89,764)	(88.0)	(754)	(671)	(89.0)
Koinadugu	-	-	-	387,289	261,416	67.5	341,987	274,206	80.2	1,620	1,306	80.6
	(62,278)	(55,725)	(89.5)	(59,121)	(50,631)	(85.6)	(72,521)	(50,631)	(70.0)	(720)	(692)	(96.1)
Kono	-	-	-	422,204	326,535	77.3	495,787	405,123	81.7	1,438	1,261	87.7
	(99,553)	(97,279)	(97.7)	(84,468)	(72,917)	(86.3)	(73,362)	(68,369)	(93.0)	(720)	(648)	(90.0)
Tonkolili	-	-	-	201,088	160,095	79.6	168,383	143,787	85.4	960	728	75.8
	(67,276)	(64,355)	(95.7)	(50,122)	(43,282)	(86.4)	(135,069)	(118,943)	(86.0)	(480)	(389)	(81.0)
**Overall**	**-**	**-**	**-**	**2,375,539**	**1,831,383**	**77.1**	**2,180,914**	**1,781,037**	**81.7**	**8,505**	**7,132**	**83.9**
	**(593,701)**	**(562,980)**	**(94.8)**	**(471,947)**	**(403,212)**	**(85.4)**	**(626,845)**	**(540,103)**	**(86.0)**	**(2,467)**	**(2,195)**	**(89.0)**

Note: Figures for SAC are shown in brackets.

In the 2011 independent monitoring, a total of 8,505 persons were interviewed, 7,132 persons recalled taking PZQ. This gave overall treatment coverage of 83.9% in people interviewed, and 89% in SAC (Table 1). Compared with the reported coverage in the same year, there was no significant difference between the reported coverage and the survey results (p > 0.05).

### Prevalence of *S. mansoni* infection

The prevalence of *S. mansoni* infection from the sentinel sites is shown in Table 2 and Figure 1. The adjusted overall prevalence in all seven districts in 2012 was 16.3% (95% CI: 14.4-18.4%). This prevalence represents a 67.2% reduction from the baseline of 49.7% (95% CI: 46.2-53.3%) in 2009 (p < 0.001). Across the seven districts, except in Bombali where a 36% reduction was seen (p < 0.05), the other six districts had a 56-98% reduction in prevalence (p < 0.001). According to the baseline endemicity level, there was no significant change in prevalence in sites where the baseline endemicity level was low (p > 0.05), while there were significant reductions (68-70%) in prevalence in sites where the baseline endemicity level was moderate or high (p < 0.001). There was a similar reduction in prevalence in boys and girls.

**Table 2:** ***S. mansoni *** **prevalence at sentinel sites in 2009 and 2012 in Sierra Leone**

	**2009**		**2012**		**Change (%)**
	**Number examined**	**Prevalence (%) (95% CI)**	**Number examined**	**Prevalence (%) (95% CI)**	
***Overall***	770	49.7 (46.2 - 53.3)	1290	16.3 (14.4 - 18.4)	−67.2
***By district***					
Bo	119	25.2 (18.3 - 33.7)	198	0.5 (0.1 - 2.8)	−98.0
Bombali	142	27.5 (20.8 - 35.3)	239	17.6 (13.3 - 22.9)	−36.0
Kailahun	90	60.0 (49.7 - 69.5)	149	16.8 (11.6 - 23.6)	−72.0
Kenema	119	60.5 (51.5 - 68.8)	199	17.1 (12.5 - 22.9)	−71.7
Koinadugu	60	83.3 (72.0 - 90.7)	100	36.0 (27.3 -45.8)	−56.8
Kono	60	65.0 (52.4 - 75.8)	101	20.8 (14.0 - 29.7)	−68.0
Tonkolili	180	35.0 (28.4 - 42.2)	304	12.2 (9.0 - 16.3)	−65.1
***By baseline endemicity***					
Low	118	4.1 (1.3 - 10.7)	201	8.7 (4.7 - 14.3)*	112.2
Moderate	294	24.7 (19.8 - 30.0)	498	7.4 (5.3 - 10.1)	−70.0
High	358	74.5 (70.1 - 78.4)	591	23.6 (20.6 - 26.9)	−68.3
***By sex***					
Boys	373	52.8 (47.7 - 57.8)	645	17.0 (14.2 - 20.0)	−67.8
Girls	396	46.7 (41.7 - 51.5)	644	15.7 (13.1 - 18.7)	−66.4

*Change in prevalence in this group is not statistically significant (p = 0.109).

### Intensity of *S. mansoni* infection

Table 3 summarizes the intensity of infection in 2009 and 2012. The adjusted mean intensity of infection in 2012 from all sentinel sites was 18.98 epg (95% CI: 11.46-26.50 epg). Compared with the baseline 134.53 epg (95% CI: 93.51-175.55 epg), there was a significant 85.9% reduction (p < 0.001). Across the seven districts, 61-99% reduction from the baseline was observed (p < 0.01). As for prevalence, there was no significant change in mean intensity of infection in sites where the baseline endemicity level was low (p > 0.05), while significant reduction by 70-88% was shown in sites where the baseline endemicity level was moderate or high (p < 0.001). A similar level of significant reduction in boys and girls was seen (both p < 0.001).

**Table 3:** ***S. mansoni *** **intensity of infection at sentinel sites in 2009 and 2012 in Sierra Leone**

	**2009**		**2012**		**Change (%)**
	**Number examined**	**Mean intensity of infection (epg) (95% CI)**	**Number examined**	**Mean intensity of infection (epg) (95% CI)**	
***Overall***	770	134.53 (93.51 - 175.55)	1290	18.98 (11.46 - 26.50)	−85.9
***By district***					
Bo	119	35.09 (17.59 - 52.59)	198	0.12 (0–0.36)	−99.7
Bombali	142	48.85 (27.67 - 70.02)	239	18.68 (5.95 - 31.41)	−61.8
Kailahun	90	194.93 (116.14 - 273.73)	149	19.33 (8.62 - 30.03)	−90.1
Kenema	119	191.60 (132.61 - 250.58)	199	25.69 (11.99 - 39.39)	−86.6
Koinadugu	60	250.80 (180.44 - 321.16)	100	42.72 (24.86 - 60.58)	−83.0
Kono	60	159.60 (87.16 - 232.04)	101	10.22 (5.66 - 14.78)	−93.6
Tonkolili	180	91.33 (57.43 - 125.24)	304	24.87 (14.94 - 34.80)	−72.8
***By baseline endemicity***					
Low	118	9.25 (0–18.56)	201	15.50 (0–34.84)*	67.6
Moderate	294	29.81 (19.14 - 40.48)	498	8.78 (2.79 - 14.76)	−70.5
High	358	226.01 (153.79 - 298.23)	591	26.41 (13.18 - 39.64)	−88.3
***By sex***					
Boys	373	133.96 (80.79 - 187.13)	645	19.30 (9.30 - 29.30)	−85.6
Girls	396	135.00 (72.98 - 197.02)	644	18.69 (7.45 - 29.94)	−86.2

*Change in intensity of infection in this group is not statistically significant (p = 0.26).

**Figure 2:**
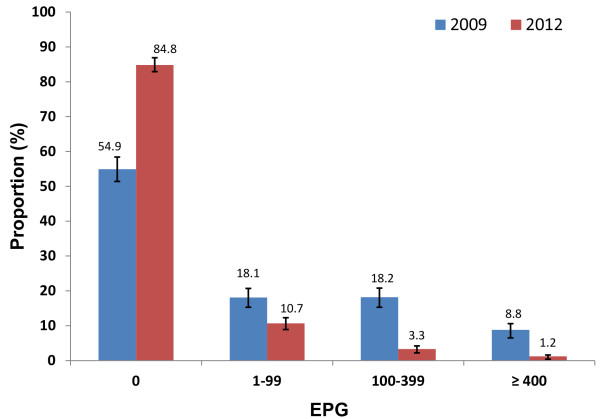
**Proportion (%) of different degrees of intensity of *S. mansoni *infection in school age children in 2009 and in 2012 in Sierra Leone.** Error bars represent the 95% confidence intervals.

At baseline in 2009, 27% school age children were moderately or heavily infected with *S.mansoni*: 8.8% heavily and 18.2% moderately. After three years of implementation of MDA, the overall proportion of children moderately or heavily infected with *S.mansoni* was significantly reduced to 4.5%: 1.2% heavily and 3.3% moderately in 2012 (Figure 2).

## Discussion

Praziquantel MDA for schistosomiasis in Sierra Leone is part of the integrated national NTD control program. According to the national baseline mapping [15-17], seven districts (Kailahun, Kono, Koinadugu, Kenema, Bo, Tonkolili and Bombali) had moderate or high levels of endemicity (prevalence ≥10% and <50%); five districts (Moyamba, Port Loko, Kambia, Pujehun and Rural Western Area) had low levels of endemicity (prevelance >0% and <10%); and two districts (Bonthe and Urban Western Area) were non-endemic. As the first phase of the control program, the PZQ distribution targets both school age children and at-risk adults for morbidity control in the seven moderately or highly endemic districts. Over the last three years, 77-95% program coverage has been achieved, which was validated by the independent monitoring survey. The high treatment coverage is important in NTD control programs using preventive chemotherapy strategy. The annual coverage achieved for schistosomiasis in Sierra Leone has surpassed the 75% coverage recommended by WHO [22]. The successful implementation of MDA in Sierra Leone was largely due to effective advocacy, social mobilization and program coordination at all levels and assisted by the introduction of independent monitoring during and after MDA. Trained independent monitors perform the ‘in process’ monitoring during the MDA to help identify MDA weaknesses, such as drug shortages, under-performing health workers, or high refusal rates within particular communities or sub-sections of communities, so that they could be reported to the NTDCP, and swift remedial action could be taken. They also perform the ‘end process’ monitoring after the MDA to collect coverage data independently and to validate the reported treatment coverage. Independent monitors also collect qualitative information, such as the misappropriation of MDA drugs, inappropriate dosage, lack of training or lack of dose poles or tally sheets.

WHO recommends conducting sentinel site surveys every 2–3 years after the start of the program implementation to assess the impact of the program [19]. As the first MDA targeted only school going children, the sentinel site survey was conducted after three years of MDA implementation when the communities and all school age children had received at least two rounds of MDA. The total cost for the survey was 13,260 US dollars, around 500 US dollars per sentinel site. The results showed that the overall prevalence of *S.mansoni* infection was significantly reduced by 67%, ranging from 36% to 98% in individual districts, and the intensity of infection was reduced by 85.9% with reductions ranging from 61% to 99% in various districts. Among the 26 sentinel sites, those with moderate or high endemicity levels at baseline showed significant reduction in both overall prevalence and mean intensity of infection. 12 sites had high prevalence (>50%) in 2009, and only two, which had 90% prevalence at baseline, still showed over 50% prevalence in 2012. It is not surprising that those sites with low endemicity level at baseline did not show significant change, in either overall prevalence or mean intensity of infection, as these were not program priority areas. However, it was noted that one of these low endemicity sites at baseline showed a significant increase in infection level over the three years (data not shown), suggesting that the national treatment strategy in low endemicity areas needs to be reviewed. As described in the previous publications, separate mapping surveys were conducted for *S. mansoni* and *S. haematobium*[15,17]. Sentinel sites were selected based on the *S. mansoni* survey and most of the sites did not have *S. haematobium* baseline data for comparison. Therefore, *S. haematobium* data were not presented in this paper. It is also noted that the current survey was conducted a few weeks after lymphatic filariasis MDA with albendazole and ivermectin, STH data collected during the sentinel site survey were not included as it would significantly overestimate the impact of the program.

After two to three years of effective MDA, the WHO estimates that <1.0% of SAC should be heavily infected (>400epg) [19]. In our results, the proportion of heavy infection was reduced from 8.8% to 1.2% after three years of implementation. The severity of morbidity due to schistosomiasis is associated with the intensity of infection, especially in early childhood [23]. It is believed that serious morbidity may have been avoided or reversed as a result of effective MDA. Similar gains have been reported by other NTDCPs in both East and West Africa in their progress towards *S. mansoni* control and elimination [24-29]. Despite the success in reducing the proportion of children heavily or moderately infected to 1.2% and 3.3% respectively, continued MDA is still needed to further bring down the prevalence and intensity of infection in school age children. It should be noted that there were 10.7% of children surveyed living with low intensity of infection in the seven districts. The consequences of morbidity due to light infections are sometimes underestimated and hence, overlooked in many control programmes. Recent studies have revealed that considerable morbidity due to diarrhea, chronic pain, under-nutrition, exercise intolerance and anaemia can result from even light infections [23,27].

WHO recommends comprehensive control measures to achieve the elimination of schistosomiasis [30-32]. In Sierra Leone, like many other countries currently implementing NTD control programs, the funding is limited to MDA, which leaves the other components of NTD control unfunded, such as water, sanitation and hygiene, and snail control. There will be a risk of recrudescence when MDA is stopped. In order to achieve the WHO target, there is a need for a full control approach which includes provision of safe water, improved personal hygiene and community sanitation and, intensified case management, veterinary public health at the human-animal interface, and vector and intermediate host control [32,33].

Sanitation is a major problem in Sierra Leone. According to the Demographic and Health Survey, conducted in 2008, 87% of the population does not have access to improved toilet facilities [34]. The provision of proper sanitation facilities: proper toilet, laundry, hand washing and shower facilities and ensuring proper personal hygiene can help to reduce the disease burden. The provision of proper toilet facilities and ensuring that these toilets are used would help to sustain the current achievement. According to the same survey, 49.2% of the population does not have access to a clean water source [34]. The absence of clean drinking water can greatly increase the spread of water borne diseases, and the provision of piped water has been shown to reduce the prevalence of schistosomiasis in SAC. In south-east Brazil, for example, research showed that SAC were 2.3 times more likely to be infected if they had no piped water in their homes, and in Zimbabwe, the prevalence of *S. mansoni* among SAC who lived on communal lands that lacked piped-water supply was 4.8% compared to 0.8% of those that had piped water on the same land [35,36].

Long-term schistosomiasis control and elimination will require health education, safe water sources for washing, and latrines in both schools and communities. In addition, behaviour change communication is needed to ensure the appropriate use of these facilities, to improve personal and environmental hygiene, and to reduce risk of re-infection. To this end, a closer collaboration among partners in relevant sectors, particularly education and water, sanitation and hygiene, is needed [37]. With the progress of the national program, the Ministry of Health and Sanitation will need to work with partners to mobilize resources to support such activities.

The World Health Assembly (WHA) resolution 65.21 calls on Member States to intensify schistosomiasis control efforts in most disease-endemic countries and to initiate interventions towards the elimination of schistosomiasis in low-transmission countries [31]. The WHO NTD control roadmap and the schistosomiasis strategic plan (2012–2020) have set the stage for the national control programs to achieve the elimination of schistosomiasis [30,32]. The results from the current survey show that Sierra Leone has made significant progress in reducing morbidity due to schistosomiasis in seven moderately or highly endemic districts, as exemplified by the significant reduction in *S. mansoni* infection and particularly, by the reduction in the intensity of infection. In line with the new elimination objectives set by WHO, the national NTDCP has reviewed the national treatment strategy and decided to target school age children for the next MDA in five coastal districts (Moyamba, Port Loko, Kambia, Pujehun and Rural Western area) where low prevalence (>0% and <10%) was found during the national baseline mapping. It is expected that a national re-assessment of the endemic situation of schistosomiasis will be conducted after 5 or 6 rounds of MDA according to the WHO recommendations [19], and the treatment strategy will be re-aligned. It is hoped that by adjusting the treatment strategy and expanding national coverage, Sierra Leone will be on course to be one of the first sub-Saharan African countries to achieve elimination of schistosomiasis as a public health problem.

## Conclusions

The national NTD control program in Sierra Leone has implemented PZQ MDA in seven moderately or highly endemic districts, targeting school age children and at-risk adults for three years. Significant progress has been made in reducing the prevalence and intensity of *S. mansoni* infection, as shown by the sentinel site survey conducted in 2012. The national program has reviewed the current treatment strategy and is expected to expand the treatment coverage to include school age children in low endemicity districts in alignment with the new national objective for the elimination of schistosomiasis. Sierra Leone is well on its way to eliminate schistosomiasis as a public health problem.

## Competing interests

The authors state they have no competing interests.

## Authors’ contributions

MH conceived the study, reviewed and revised the paper. SS managed and implemented the national NTD control program. JP planned and coordinated the field data collection and drafted the paper. FMM, AC, MS coordinated the MDA activities. JP and MSB collected the field data, performed the laboratory investigations and the initial data analysis. YZ trained the technicians, performed final data analysis and revised the paper. All authors read and approved the final manuscript.
